# Startup Ethics: Ethically Responsible Conduct of Scientists and Engineers at Theranos

**DOI:** 10.1007/s11948-022-00393-2

**Published:** 2022-08-30

**Authors:** Robert E. McGinn

**Affiliations:** 1 Department of Management Science and Engineering, School of Engineering, Huang Engineering Center, Stanford University, 475 Via Ortega, Stanford, CA 94305 USA; 2 Department of Biochemistry and Biophysics, School of Medicine, Lead Ethics Investigator, Center for Cellular Construction, University of California, 600 16th Street, Box 2240, San Francisco, CA 94158 USA

**Keywords:** Theranos, Technical startups, Startup ethics, Fundamental ethical responsibilities of scientists and engineers, Ethically responsible conduct, Harm-prevention strategies

## Abstract

Studies of ethical challenges that can confront practicing scientists and engineers in the entrepreneurial stage of the overarching research-and-innovation process are virtually non-existent. This paper explores ethical challenges that arose at a specific entrepreneurial startup: Theranos, the defunct blood-testing company. The fundamental ethical responsibilities of scientists and engineers (FERSEs) offer a framework useful for evaluating the conduct of practicing scientists and engineers from an ethical responsibility perspective. Questionable conduct by Theranos’s former top managers has been widely discussed. However, the fact that a number of Theranos scientists and engineers responded to ethical challenges in several phases of the innovation/entrepreneurial stage with ethically responsible conduct has gone largely unrecognized. Ten mini cases involving these practitioners are discussed. Their deeds reflect different harm-prevention strategies. The Theranos case suggests several ethics-related takeaways for scientists and engineers who work or may work in technical startups. Familiarity with the FERSEs and knowledge of the ethical challenges, ethically responsible conduct, and harm-prevention strategies exhibited in the Theranos case provide valuable intellectual resources for startup scientists and engineers who aspire to be ethically responsible professionals.

## Introduction

The literature on ethical issues and challenges in the research stage of the overarching research-and-innovation process is substantial.^1^ However, scholarly interest in such issues and challenges in the entrepreneurial stage of that process has been minimal. While some ethics studies related to entrepreneurial endeavor have appeared,^2^ accounts of ethical issues and challenges that can face practicing scientists and engineers working in technical startups have been rare. To shed light on such issues and challenges and how technical practitioners react to them, this paper explores the conduct of selected scientists and engineers employed by a specific technical startup: the defunct blood-testing company, Theranos.

Most writing about the Theranos case has focused on conduct by the firm’s CEO, Elizabeth Holmes, and its COO, Ramesh Balwani. For example, John Carreyrou’s book, *Bad Blood: Secrets and Lies in a Silicon Valley Startup*^3^ (BB), explores their conduct in detail. However, that work also relates deeds of many practicing scientists and engineers employed by the company.  The latter feature makes BB an excellent resource for the present study.^4^

After an overview of the Theranos episode, a framework useful for assessing the conduct of scientists and engineers from an ethical responsibility perspective is presented. Drawing on it, the conduct of 10 Theranos scientists and engineers is discussed, focusing exclusively on conduct that is ethically *responsible*.^5^ Strategies are then identified that technical practitioners utilized in striving to be ethically responsible. Finally, ethics-related takeaways pertinent to startup scientists and engineers are proposed and a general conclusion is reached.

## The Theranos Episode: An Overview^6^

“[A]bsolutely terrified of giving blood”^7^ since childhood, Elizabeth Holmes planned to succeed by transforming the blood-testing industry, eliminating venipuncture and syringes. Her idea was to extract a few drops of patient blood by finger-stick, put the drops into a small capsule, which she dubbed a “nanotainer,” transfer the nanotainer blood to a credit-card-sized plastic “cartridge,” insert the cartridge into a desktop-computer-sized programmable device called a “reader,” automatically perform one or more tests on the sample, and quickly provide results. Her professed goal was to save lives by determining the levels of health-compromising substances in the patient’s blood well before health problems reached a critical or irreversible stage.

Holmes entered Stanford University as a freshman in September 2002. She spent the following summer at the Genome Institute of Singapore, where she tested for SARs-CoV-1 by “collecting blood samples with a syringe and mucus with nasal swabs.”^8^ Upon returning home, she drew up “a patent application for an arm patch that would simultaneously diagnose medical conditions and treat them.”^9^ After her patch idea was rejected as infeasible by a Stanford professor of medicine,^10^ Holmes approached Channing Robertson, a Stanford professor of chemical engineering. He granted her request to work as an assistant in his research lab with Ph.D. students and encouraged her to pursue her dream.^11^ Holmes incorporated “Theranos” a month after she dropped out of Stanford in March 2004.^12^ She raised considerable startup and sustaining capital^13^ and recruited a distinguished Board of Directors.^14^

Holmes’s initial idea was to develop a wearable, smart patch, able to draw blood, perform tests, modify drug dose levels, and send information to doctors wirelessly. However, the patch idea morphed into a series of developmental prototypes: the “Theranos 1.0,” multiple versions of the “Edison,” the “4S,” and the “miniLab.” The Edison could only do immunoassays.^15^ However, Holmes insisted that the elusive miniLab prototype be able to carry out tests from the four major types of blood assays: immunoassays, general chemistry assays, hematology assays, and assays that rely on copying DNA strands.^16^

As a clinical lab, Theranos had to meet federal regulatory requirements to be certified to carry out blood tests and provide results to human patients.^17^ Each assay that the firm claimed its prototype could carry out accurately had to be validated. To do so, the prototype had to meet “quality control” (QC) standards, indicating that the test carried out with it was accurate. This involved processing a sample of blood plasma sent to it by an accredited agency that contained a level of the analyte being tested known only to the agency. Only if the result returned to the agency was within two standard deviations of the known level would the quality-control test be deemed successful.^18^

Theranos also had to show that tests carried out multiple times on the same sample had a “coefficient of variation” (CV) of less than 10 percent, indicating that the test was relatively consistent.^19^

Finally, like all clinical labs without waivers, the Theranos clinical lab had to undergo thrice-yearly “proficiency testing” (PT) to ensure that its testing procedures were accurate across a wide range of analytes, indicating that the lab was reliable. An accredited agency would send the lab being evaluated a set of samples of preserved blood plasma with levels of various analytes known only by the agency. The lab would test the samples, using the technologies normally used in its lab testing, and send the results back to the agency to be compared with ones submitted by peer laboratories, hopefully showing that the lab was not an outlier. U.S. clinical laboratories “must demonstrate *successful* PT performance to remain in good standing” and thus be able to operate.^20^

Since its founding, Theranos operated as a R&D unit, testing the blood of volunteer donors and giving employees small cash incentives.^21^ But in 2010, Holmes decided to take the company commercial. In 2012, Theranos signed a lucrative contract with Walgreens to set up “Wellness Centers”^22^ in Walgreens stores equipped with Theranos's blood-testing system. After repeated delays, Walgreens opened its first Theranos Wellness Center in September 2013, eventually reaching 45, the bulk in Arizona Walgreens stores.^23^ However, given the miniLab’s continuing reliability problems, the CEO and COO decided that the older Edison prototype should be used to process patient samples.^24^

By 2014, several Theranos employees had become sufficiently upset over how the company was operating that, when their expressed concerns went unaddressed, they resigned or were fired. Eventually, through a complex chain of events, two critical developments occurred. In September 2015, a former Theranos employee contacted the Centers for Medicare and Medicaid Services (CMS), the federal regulator of clinical labs.^25^ The employee alleged a range of questionable practices by Theranos. This led senior CMS field inspectors to conduct a surprise multi-day inspection of the Theranos clinical lab in Newark, California, in late September, with a return visit two months later.^26^ CMS found many violations, most of which remained uncorrected.^27^ In October 2015, the *Wall Street Journal* published an article containing allegations of problematic Theranos lab practices.^28^

Although Theranos voided tens of thousands of blood-test results in its attempt to come into compliance with CMS standards,^29^ in July 2016 the agency barred Theranos from running a clinical laboratory.^30^ Lawsuits against Theranos followed, resulting in the return of many millions of dollars to investors and the recovery of $4.65 million in testing fees paid by 75,217 Arizona Walgreens clients.^31^ Walgreens closed 40 Wellness Centers in Arizona in June 2016^32^ and Holmes announced that Theranos would close its clinical labs in Arizona and California in October 2016.^33^ Theranos was compelled to dissolve in September 2018.^34^

In March 2018, the U.S. Securities and Exchange Commission (SEC) charged Holmes and Balwani with defrauding investors.^35^ In June 2018, the U.S. Attorney for the Northern District of California charged them with two counts of conspiracy to commit wire fraud and nine counts of wire fraud.^36^ Holmes’s trial began on September 8, 2021.^37^ On January 3, 2022, she was convicted of three counts of defrauding investors and one count of conspiring to defraud investors.^38^ Balwani’s trial began on March 22, 2022.^39^ On July 7, 2022, he was convicted of conspiracy to defraud Theranos investors, conspiracy to defraud Theranos paying patients, and ten counts of wire fraud against specific Theranos investors and patients.^40^

## The Fundamental Ethical Responsibilities of Scientists and Engineers

Before turning to the conduct of selected Theranos scientists and engineers, brief comments on the framework used in evaluating that conduct are in order. To merit serious consideration, claims about whether a scientist’s or engineer’s conduct at work is ethically responsible must be anchored in something more compelling than subjective judgment, e.g., some kind of ethical framework.^41^ A framework that has proven useful in this regard involves the author’s “Fundamental Ethical Responsibilities of Scientists and Engineers” (FERSEs).^42^ To the extent that a scientist’s or engineer’s conduct is in accord with/violates the FERSEs, that counts in favor of/against deeming it ethically responsible.^43^

Medical doctors are widely held to have a fundamental ethical responsibility to their patients: “to do no harm.” In contrast, few recognize that scientists and engineers also have “patients” – those affected by their professional actions – to whom they too have the overarching fundamental ethical responsibility to “do no harm.” “Do no harm” means more than simply not *causing* harm to others through one’s work. It also involves trying to *prevent* certain harms and trying to *alert* those at risk of incurring certain harms that they are vulnerable. Unpacking “do no harm” for scientists and engineers yields the following fundamental ethical responsibilities:

1. ….to not cause harm or create an unreasonable risk of harm to others (or to public welfare or the public interest) through their work (FERSE1).
2. ….to try to prevent harm or an unreasonable risk of harm to others (or to public welfare or the public interest) from their work or work of others with which they are familiar and about which they are technically knowledgeable (FERSE2).^44^
3. ….to try to alert and inform individuals and segments of the public at significant risk of being harmed by their work or work of others with which they are familiar and about which they are technically knowledgeable, that they are vulnerable to that risk (FERSE3).

In addition, any scientist or engineer who is employed by an organization or who works for a client has an additional fundamental ethical responsibility…

4 ….to do her/his best to serve the legitimate interests of the employer/client (FERSE4).^45^

Thus formulated, the FERSEs need several clarifications. First, besides physical injury, disease, and financial loss, “harm” also encompasses significant societal and psychological damage. Second, “causing” harm involves not just foreground factors that precipitate or trigger a harmful outcome, but background enabling, facilitating, stimulative, and other kinds of contributory causal factors. Third, “others” includes not just individual affected humans, but public welfare, the public interest, and certain affected non-human sentient beings, such as dogs, pigs, and monkeys.^46^ Fourth, FERSE1 can be violated not only by deliberate acts of commission but also by negligent acts of omission. Fifth, in FERSE2 and FERSE3, the fundamental ethical responsibilities asserted are not to successfully prevent harm or an unreasonable risk of harm or to successfully alert about vulnerability to harm, but to *try* to do so. Sixth, in FERSE4, scientists and engineers do not have an ethical responsibility to further *all* interests of their employers or clients; only those of them that are *legitimate.* 47

## Ethically Responsible Conduct of Theranos Scientists and Engineers

In responding to the ethical issues and challenges they faced, a number of Theranos scientists and engineers acted commendably from an ethical-responsibility point of view. Ten illustrative mini cases (**MC1-10**) follow.^48^

**MC1**. Having worked at Apple and Adobe as a product designer and manager, Ana Arriola became Theranos’s chief design architect in August 2005.^49^ She learned from an engineer-colleague about a pilot study Theranos was conducting for Pfizer in Tennessee. It involved home use by terminally ill cancer patients of the Theranos 1.0 prototype blood analyzer. Arriola feared the human subjects might be used as guinea pigs to test an unreliable device, something she believed would cross an ethical line.^50^

Arriola confronted Holmes about the Theranos 1.0 system’s problems and their potential human consequences. She urged her to pause the pilot study until the reliability problems were resolved. Holmes refused, citing demand for the blood-testing system by large drug companies. The CEO told Arriola to reflect on whether Theranos was the right place for her.^51^ She did – and resigned forthwith. Arriola merits respect for heeding her conscience, confronting the CEO about the risks involved, and resigning because she believed that, in Carreyrou’s words, proceeding with the pilot study “wasn’t the right thing to do.”^52^

An ethical issue that Arriola faced was whether to continue working for a company she believed was prioritizing profit over not putting human patients at risk with its work, even if she was not part of the group conducting the pilot study. Under FERSE2, scientists and engineers have an ethical responsibility to try to prevent harm or an unreasonable risk of harm to others (or to public welfare or the public interest) from their work or work of others with which they are familiar and about which they are technically knowledgeable. To the extent that Arriola was familiar with the phenomenon of users being put at risk by using unreliable products and was reasonably technically knowledgeable about the pilot study, FERSE2 applied to her in this situation. By confronting Holmes about the risky study and urging her to pause it until the prototype’s reliability improved, Arriola was trying to prevent harm and unreasonable risks of harm to humans.  In short, she tried to do what FERSE2 requires of an ethically responsible technical professional.

**MC2**. Engineer Aaron Moore studied at Stanford and MIT and was interested in microfluidics.^53^ Based on his observations at work, he was concerned about the effects of what he viewed as poor communication between Theranos’s engineering and chemistry groups, not just organizationally but in terms of the downstream consequences of such communication for humans. The engineering and chemistry groups were conducting their respective tests on the parts of the Theranos 1.0 system for which they were responsible. However, no one was carrying out overall system tests.^54^ That troubled Moore, even after the Theranos 1.0 gave way to the first version of the Edison prototype.

Mindful of the pilot project for Pfizer, Moore^55^ decided to conduct informal human-factors field research on the Edison system, including lancets to draw blood and small syringes to transfer it into cartridges.^56^ He concluded it was naive to think older patients could operate the system flawlessly in their homes each day, something seemingly taken for granted in the pilot study.^57^ To Moore, the Edison’s poor usability compromised its reliability and increased the risk it posed. Moore told Holmes and his engineer-boss his conclusions, but the importance of good usability seems not to have been a priority for Theranos management. When Moore complained repeatedly to his boss about the Edison’s unreliability, he was told,  “Go find a place where you can be a big fish in a small pond.”^58^ Moore left Theranos in June 2008.^59^

Carrying out a human-factors field study on the Edison system to probe a neglected source of risk in the pilot project, and calling management’s attention to the finding that the prototype’s usability was lacking, something Moore believed increased the risk the system posed to patients testing themselves at home, is conduct well aligned with FERSE2. Taking a systemic and field-use-sensitive approach to risk and reliability, as Moore did, is something about which all would-be ethically responsible engineers in startups should be mindful.

**MC3**. After working at Apple, software engineer Justin Maxwell came to Theranos to work on the design of Edison software and other parts of the system with which humans would interact. The siloization of information he observed at Theranos distressed him, for that practice impeded fruitful interdepartmental communication.^60^ He was also perturbed by the frequent firings and intrusive IT surveillance of employees at Theranos, and by the prevalence of dishonesty at the firm, including the presence of employees whom he viewed as “yes men” to the CEO.^61^

At first glance, these phenomena do not appear to raise ethical issues or challenges. But, given what he observed at Theranos and what he viewed as the current and likely future negative effects of these phenomena on the company, Maxwell faced a difficult ethics-related choice: remain silent about his concerns or bring them forward. It is, however, difficult to reconcile someone in his position remaining silent about what he observed with respecting FERSE4’s notion that the employed scientist or engineer has an ethical responsibility to do her/his best to serve the legitimate interests of her/his employer. For being made aware of company practices that impair employee productivity and impede employee retention is a legitimate employer interest.

It might be argued that the only legitimate employer interests that the employed scientist or engineer has an ethical responsibility to serve are purely technical ones. However, the legitimate employer interest of getting the best technical work from its technical employees sometimes involves their identifying organizational features that are thwarting such work, something they may be especially or uniquely well positioned to do.

Maxwell chose to bring his concerns forward in a novel way. He emailed a letter of resignation to the CEO. In it, he urged her to read specific managerial books that explored the negative effects on employees of disturbing corporate work practices, and argued that what he called Theranos’s “cultural disease” needed to be addressed along with its technical challenges.^62^ Since problematic organizational attributes can engender significant risks of harm to workers and customers as much as flawed technical systems can, Maxwell’s conduct in alerting and encouraging Holmes to address the problems he discerned was ethically responsible under FERSE2 and FERSE4.^63^

Heavy managerial pressure on a startup’s technical professionals to work rapidly, incessantly, and relentlessly to get its innovative product to market as quickly as possible often puts startup scientists and engineers in an ethical bind. Such pressure may not only elicit irresponsible shortcutting behavior, but also result in management giving harm- and risk-engendering organizational features and practices short shrift when scientists or engineers trying to be ethically responsible express concerns about their workplace consequences.

**MC4.** Tyler Shultz initially majored in mechanical engineering at Stanford but switched to biology as a senior.^64^ He joined Theranos in 2013 and worked in the immunoassay group.

A blood test is normally considered precise if the results of multiple test runs vary by less than 10 percent. Shultz observed that in some cases Theranos claimed CVs of less than 10 percent even though lab data indicated otherwise.^65^ One assay validation experiment Shultz worked on was a blood test for syphilis. The Edison detected 65% of the positive samples on the first run, 80% on the second. This perturbed Shultz:

If a hundred people who had syphilis came and got tested on the Theranos devices, [Theranos] would only tell 65 of them that they had syphilis, and [it] would tell the other 35, ‘You’re healthy, no need for medical intervention.’ So, if people are testing themselves for syphilis using Theranos, there’s going to be a lot more syphilis in this world.^66^

These percentages notwithstanding, in its validation report for that test Theranos claimed the Edison had recognized 95% of the samples infected with syphilis.^67^

Shultz came to believe that Theranos was also misleading about other tests, e.g., one to measure the level of vitamin D. Results of vitamin D tests of samples processed on one of the firm’s purchased commercial analyzers differed significantly from those yielded by the Edison.^68^ Yet the Edison vitamin D test was “cleared for use in the clinical lab on live patient samples.”^69^

At his request, Shultz met with Holmes about the accuracy of Edison tests. She suggested he talk with company Vice President Daniel Young to discuss his concerns that Theranos validation reports were claiming more precision for tests than he had seen in the data. Young eventually acknowledged that "…sometimes Elizabeth exaggerates in an interview setting."^70^ However, exaggerated performance claims made during interviews cannot be legitimately incorporated into formal blood-test validation reports.

Besides concerns about the precision of some company blood tests and the integrity of its assay validation reports, Shultz had a problem with Theranos proficiency testing. Theranos’s clinical lab directors had ordered that certain PT samples be divided and tested on both other companies’ commercial analyzers and on Theranos’s Edison prototype.^71^ The results obtained differed substantially, especially for vitamin D. What troubled Shultz was that in its PT reports Theranos used only the results obtained from the commercial machines it had purchased, not the results from the proprietary Edison system which was often being used to carry out patient tests.^72^

Rebuffed by top management in his initial attempt to prevent harm and unreasonable risks of harm, Shultz took his PT concerns extramural. Using a pseudonym and without mentioning Theranos by name, he wrote to an official with the Clinical Laboratory Evaluation Program at the New York State Dept of Health about Theranos’s PT practices. The official replied that what the company was doing was “a form of PT cheating.”^73^ Shultz wrote again to Holmes about Theranos’s PT: “I just feel a responsibility to tell you what I see so we can work toward solutions.”^74^ Balwani replied to that email, severely reprimanding Shultz, who then resigned.^75^

Although Theranos threatened him with a lawsuit, and despite his parents incurring major legal expenses to assist him, Shultz agreed to talk with and provide relevant documents to Carreyrou who was doing research for his 2015 article about Theranos. While FERSE2 states that scientists and engineers have a fundamental ethical responsibility to try to prevent harm or unreasonable risks of harm to others from certain work, it does not automatically imply an ethical responsibility to engage in public whistleblowing.^76^ In this instance, however, it was only after his serious safety concerns were dismissed twice by top management that Shultz, with ample insider knowledge and pertinent documentation, took his effort to prevent serious risks of harm extramural by indirectly going public. His conduct was highly ethically responsible under FERSE2.

**MC5.** After graduating from U. C. Berkeley with majors in biology and linguistics, Erika Cheung joined Theranos as a lab associate in October 2013. She began in the immunoassay group, validating tests for various analytes.^77^ Subsequently she moved to the part of the clinical lab where researchers were working with Edisons.^78^

On one occasion, when an order came in for a vitamin D test, Cheung “ran a quality control test on the Edison before testing the patient sample.”^79^ The goal was to see whether the Edison was accurate by running a sample with a known level of analyte and comparing the known level with the test result. If the result was more than two standard deviations higher or lower than the known value, the machine would be viewed as having failed the QC test. The Edison failed the QC check twice. When Cheung inquired what to do, an employee from the R & D lab arrived, deleted two data points the employee treated as outliers, tested the patient sample, and sent out a result.^80^ Cheung was shocked. She testified at Holmes’s trial that the practice of deleting outliar data points to resolve quality-control failures happened frequently at Theranos. Moreover, she stated, at Theranos there was no definition of an “outliar” and “no point person…to determine what an outliar was.”^81^

The main reason the data deletion episode upset Cheung was that receiving and acting on a flawed test result can jeopardize a patient’s health. At Holmes’s trial, Cheung rejected any attempt to rationalize inaccuracies in Theranos tests by claiming they cost less than conventional blood tests:

Just because [Theranos tests] might be cheaper doesn’t mean you should give people false information about their health status. It shouldn’t be the case [that] because you pay less [for a test], you get a less quality result.^82^

After the data deletion episode, the lab’s assay validation team cleared an Edison-based hepatitis C test, notwithstanding expired reagents and overdue recalibration of the Edisons.^83^ Given the infectious nature of hepatitis, Cheung refused to run the cleared hepatitis C test on a patient sample with an unreliable Edison machine. Instead, after talking with the lab codirector, she ran the test with a commercially available hepatitis kit. When the COO learned of this workaround, he became angry. When she raised her concern about lab quality-control failures with him,^84^ he criticized her and told her “You need to tell me whether you want to work here or not.”^85^ Concluding that her concerns were not being taken seriously, Cheung resigned in April 2014.^86^

While Cheung, like Shultz, spoke to Carreyrou after she resigned, she also took a different tack. She contacted the Centers for Medicare and Medicaid Services (CMS) by email and shared her concerns about the lab in detail.^87^ This quickly led to a surprise CMS inspection of Theranos’s Newark lab. Many problems were discovered. The firm was eventually banned from the clinical lab business.^88^ Theranos threatened in June 2015 to sue Cheung for allegedly violating a nondisclosure agreement she had signed upon resigning by speaking with Carreyrou.

In light of her commitment to conducting rigorous quality-control checks of the Edison prior to testing patient samples, opposition to data falsification and sending patients flawed test results, calling top management’s attention to Edison reliability problems, speaking with Carreyrou, and testifying about her Theranos experience at Holmes’s trial, Erika Cheung’s conduct was impressively ethically responsible under FERSE1 and FERSE2.^89^

**MC6**. In 2012–13, Diana Dupuy was a certified clinical laboratory scientist (CLS) and Clinical Laboratory Improvement Amendments (CLIA) consultant at Theranos’s clinical laboratory in Palo Alto. She specialized in blood transfusion, was knowledgeable about government regulations for clinical labs, and followed lab rules fastidiously. Dupuy observed in her lab what she regarded as poor or illicit work practices on the part of an inexperienced, sloppy, and poorly trained CLS. She believed his actions were making lab blood test results inaccurate, hence unreliable and risky. After talking with him, Dupuy sent emails documenting his violations to the lab head and the COO.^90^ They fell on deaf ears. Per Carreyrou, the COO fired Dupuy, ostensibly for calling attention to the fact that a vendor had stopped filling lab purchase orders because of unpaid bills.^91^

In scrupulously following lab rules and regulatory procedures, Dupuy acted in accord with FERSE1. In documenting laboratory practices that she believed jeopardized blood-test accuracy, and in reporting them to her lab’s director and the company COO, Dupuy acted in accord with FERSE2. In short, she avoided causing unreasonable risks of harm to patients by carefully following standard laboratory test practices, and tried to prevent unreasonable risks of harm by documenting and reporting sloppy and illicit lab practices that she believed were creating them. Since her conduct was in accord with FERSE1 and FERSE2, it qualifies as ethically responsible.

**MC7**. From 2005–2010, biochemist Dr. Ian Gibbons led or co-led Theranos’s chemistry group and helped design blood tests. But “the data he collected suggested” that the blood tests he and other chemists had designed rarely “perform[ed] as accurately [when done] inside the Theranos devices as they did on the lab bench.”^92^ Gibbons worried about these disparities in accuracy and tried to do something about them.

He seems to have recognized that the risk a product – here, a test – poses depends not just on how it works in the idealized, controlled environment of the research lab, but also on how it performs in concrete contexts of use in the field. In interactions with Theranos engineers, Gibbons argued against accepting lower standards of accuracy for Edison-run field tests. Not to do so would have been to acquiesce in commercial testing of customers that was less accurate, hence riskier, than when done in the lab. Per Carreyrou, Gibbons “butted heads” with the engineer-leader of the Edison development team over the tension between adhering to undiminished test-accuracy standards and advancing the pressured prototype-development project.^93^ His efforts to prevent unreasonable risks of harm were admirably ethically responsible under FERSE2.

**MC8**. Surekha Gangakhedkar^94^ was manager of assay systems at Theranos from July 2009 to August 2013. She knew some Edison-based tests were unreliable.^95^ In 2013,  talk at the company that the commercial processing of patient samples with the latest Edison machine was to begin shortly troubled her. She viewed the launch of testing at Walgreens as, in Carreyrou’s words, an “unauthorized research experiment” on human subjects, “something she couldn’t live with.”^96^ Gangakhedkar urged Holmes to delay starting Walgreens testing until the prototype was reliable, but to no avail. Deeming testing customers with the Edison in its current state of development too risky and ethically unacceptable, she resigned in September 2013. This was before Walgreens testing began, but after she had had multiple meetings with Holmes in which she underscored accuracy problems with various Edison-based tests poised for commercial use.^97^

Gangakhedkar was confronted with an ethical issue that often challenges technical professionals in medical startups: when is it ethically acceptable to take – or be involved with taking – a R&D venture commercial? To her ethical credit, she made a precautionary case directly to Holmes for delaying the start of testing at Walgreens pharmacies until the Edison’s reliability problems were resolved. This suggests she regarded delaying the start of testing until reliability issues were resolved as a requirement for the transition from R&D to commercial operations to be ethically acceptable and for participating in it to be ethically responsible.

At Holmes’s trial, Gangakhedkar testified that the CEO had pressured her to validate Edison-based blood tests for clinical use when it was clear to her that they were unreliable.^98^ She also testified that Holmes knew that the newest version of the Edison machines that were about to be used in Walgreens testing still had reliability issues, and that when Gangakhedkar urged her to delay the launch, she declined, citing the start date she had given to Walgreens:

I raised the concerns on how she plans to launch with the Edison 3.0s; we had seen within the last few days that they continued to have [reliability] issues. At the time she mentioned she had promised to deliver to the customers, and she didn’t have much of a choice [but] to go along with the launch.^99^

Gangakhedkar’s efforts to persuade Holmes to delay the launch of testing at Walgreens out of concern for patient safety, the fact that she spoke with government investigators in 2016 and later with Carreyrou to aid their investigations of Theranos,^100^ and her court testimony that Holmes knew the Edison machines were unreliable but was determined to proceed with the launch and had pressured her to validate unreliable tests, show that her conduct in this episode was highly ethically responsible under FERSE2.

The Theranos case vividly illustrates that a technical startup’s dire need for money to continue to operate, coupled with its CEO’s related decision to commit to and stick with a premature date for transition to commercial operations, can pose a major ethical challenge to startup scientists and engineers who take being an ethically responsible technical professional seriously.

**MC9**. From April 2013 to December 2014, Adam Rosendorff, M.D.,^101^ served as director, then codirector, of Theranos’s clinical lab.^102^ His distress at what was happening at Theranos increased over time, including how the company went about doing its required proficiency testing, the inaccuracy of blood tests done with the Edison, and the fact that although he was still responsible for the integrity of clinical lab operations, he was no longer being shown quality-control data.^103^ Rosendorff conveyed his concerns about various Theranos activities, including proficiency testing, in numerous emails to Holmes and Balwani, and stored copies of many such exchanges in his personal Gmail account.^104^ At Holmes’s trial, he testified he had struggled in vain for months to get Theranos management, including Holmes, to address problems with “test results and to implement a legally required process to check the accuracy of the lab’s equipment and practices.”^105^

Rosendorff testified that, as director or codirector of the clinical lab, he was often called upon to “come up with reasons other than test performance” to explain unusual results to patients’ doctors.^106^ In fact, “At Theranos, I felt pressured to defend the company’s results to physicians.”^107^ Holmes’s brother, also a Theranos employee, asked Rosendorff to address a new skeptical query from a doctor about the accuracy and reliability of a Walgreens test result. Although he had done so previously, this time Rosendorff refused and resigned.^108^

After he resigned, Rosendorff shared his concerns about Theranos with Richard Fuisz, a doctor and medical inventor who had been sued by the company for intellectual property theft. He told Fuisz, “You and I took the Hippocratic Oath, which is to first do no harm. Theranos is putting people in harm’s way.”^109^ Eventually, he agreed to talk with Carreyrou, to whom he gave a detailed account of problematic activities at Theranos and with whom he shared some of the email correspondence he and his codirector had had with Balwani and Vice President Daniel Young about proficiency testing.^110^ At Holmes’s trial, Rosendorff testified that “Even months after I left the company, I felt obligated from a moral and ethical perspective to alert the public” about the company’s inaccurate test results. He testified that when Carreyrou called him, he used that opportunity to do so indirectly.^111^

Rosendorff’s refusal to vouch for the test result as Holmes’s brother had requested, his sharing of information and documents with Carreyrou, and his public testimony at Holmes’s trial were well aligned with the FERSE2 ethical responsibility to try to prevent harm and unreasonable risks of harm – and with the FERSE3 ethical responsibility to try to “alert and inform” those at risk of harm, viz., many past and future Theranos blood test clients.

**MC10**. Biomedical scientist Dr. Mark Pandori became codirector of Theranos's clinical lab in December 2013. However, he resigned only five months later, on the day the COO “summarily rejected” his request that Holmes run claims about prototype testing capabilities past the lab codirectors for vetting before releasing them to the press.^112^

Making inflated medical-test-capability claims in the media can engender serious risks of harm to patients, since they may prompt misguided medical decisions by patients who assume the company claims are true. Thus, public hyperbole about medical test accuracy falls within the purview of ethics. Whether to acquiesce in or attempt to deter or thwart the release of exaggerated claims about test accuracy is a bona fide ethical issue, one that can face industrial scientists in positions like Pandori’s. Through his bold request to top management, Pandori was effectively trying to prevent the release of claims likely to engender heightened risks of harm to potential test patients. For that reason, his precautionary request qualifies as ethically responsible conduct under FERSE2.

## V. Strategies of Harm Prevention

Becoming familiar with the ethical challenges Theranos scientists and engineers faced, as well as with their conduct in response to those challenges, could be extremely valuable to scientists and engineers. Those familiar with the mini cases would acquire knowledge of various kinds of problematic situations they might encounter in startups and of options open to them, with their respective benefits, costs, and risks. All the ethical challenges that arose involved, directly or indirectly, potential harm or significant risks of harm to humans from company activities, decisions, and practices. Wanting to be ethically responsible, each mini-case scientist or engineer opted for one or more strategies for combatting harm. Familiarity with the following seven harm-prevention strategies (**S1-S7**) utilized by the mini-case technical practitioners could also be potentially useful to startup scientists and engineers.

**S1**. **Persuasion**. Eight Theranos technical practitioners in the mini cases took their concerns to an executive or manager with decision-making authority, usually the CEO or COO. They tried to persuade her/him to take steps to stop existing harms, or to avoid causing new harms or creating new unreasonable risks of harm.

Two technical practitioners argued for pauses in a pilot project they deemed too risky (MC1 and MC2); one invoked medical-ethical considerations, the other disturbing new factual findings. Three others tried to persuade top managers there were serious flaws in Theranos blood tests, assay validations, quality control tests, and proficiency-testing practices (MC4, MC5, MC9). Another tried to persuade the CEO to delay launching a major new initiative that the practitioner deemed premature until the Edison’s risk was mitigated by further R&D work (MC8). Yet another, hoping to thwart the diffusion of hyperbolic claims about the capability of Theranos blood tests, tried to persuade the COO to change the process by which technical  communications released to the public were vetted (MC10). Finally, another tried to persuade the CEO to modify features of corporate culture and organizational structure that he believed were hindering effective interdepartmental and interpersonal communication, undermining employee morale and productivity (MC3).

**S2**. **Resignation**. Eight Theranos scientists and engineers in the mini cases resigned their positions (MC1-5, MC8-10). This reflects the fact that most had previously tried, unsuccessfully, to effect change from within by another strategy, usually persuasion. They eventually concluded that the only way of avoiding having to choose between being an employee who remained silent about company activities, decisions, and practices they deemed ethically unacceptable, and being an employee who, for continuing to voice her/his concerns, would probably be fired for being ‘troublesome’ or ‘uncooperative,’ was to resign.^113^ One Theranos engineer coupled his resignation with an eleventh-hour attempt at persuasion: in his resignation email he tried to persuade the CEO that certain features of corporate culture were undermining important company interests and needed to be changed. (MC3).

**S3**. **Disclosure**. Four mini-case technical practitioners who resigned opted to provide, directly or indirectly, evidence of corporate malfeasance to external parties whom they hoped might be able to stop the risky activities they had failed to stop internally. In three cases, the disclosure recipient was the same investigative journalist (MC4, MC8, MC9); in the other, an official of a cognizant government regulatory agency (MC5). Lumping these disclosures together as acts of “public whistleblowing” obscures noteworthy differences in the senses in which they were “public,” in the degrees to which they were document-supported, and in what the recipients did to address the allegations of misconduct they received.

**S4**. **Testimony**. Three mini-case technical professionals – Erika Cheung, Surekha Gangakhedkar, and Adam Rosendorff – opted to testify in a public judicial proceeding – Holmes’s trial – about what they viewed as managerial misconduct, presumably partly to deter the recurrence of harm (M5, M8, M9). In effect, these practitioners utilized the same four-element  strategic sequence: intramural persuasion→positional resignation→extramural disclosure→public testimony. Their harm-prevention efforts warrant designating them as exemplary ethically responsible technical professionals.

**S5**. **Documentation of Problematic Lab Practices**. One Theranos clinical laboratory scientist was concerned about the accuracy of test results generated in her lab. She believed that some practices of an inadequately trained technician were so risky and illicit that they jeopardized the accuracy of lab tests. She sent emails to the lab head and the COO in which she documented the practices in hopes of deterring them (MC6).

**S6**. **Opposition to Weakening Test Standards**. A Theranos senior scientist worried that data he had collected showed that the accuracy of blood tests he and his colleagues had developed in the research lab was often significantly lower when executed with the Edison in the field. He made a case to the head Edison development engineer that lowering standards of test accuracy would pose a heightened risk of harm to humans and opposed accepting lower test standards because of pressure from top management to get the Edison prototype up and running (MC7).

**S7**. **Refusal to Carry Out an Assigned Task**. Prior to resigning and opting for external disclosure, two mini-case scientists reached points in their work where they refused to perform assigned tasks because they deemed them too risky. One refused to conduct a hepatitis C test on a patient sample using the Edison (MC5), while the other refused a request by the CEO’s brother to vouch for the accuracy of an unusual Edison blood-test result to a Walgreens customer’s doctor (MC9).

## Takeaways

The Theranos case suggests several ethics-related lessons for potential startup scientists and engineers. It would be prudent for scientists and engineters who work for, or are considering working for, a technical startup and who value ethically responsible professional practice to heed the following cautions:

1. Avoid participating or acquiescing in deceptive product-validation practices that are ordered, encouraged, or tolerated by management.
2. Be wary of premature transitions from R&D work to commercial operations, especially if contract-stipulated launch deadlines, monetary incentives, or serious corporate economic problems are involved.
3. Be suspicious of moves to truncate a prototype’s design/build/test process.
4. Oppose attempts to meet government regulations on a firm’s product or process by “gaming the system” to obtain or retain an operating license.
5. Oppose putting a novel prototype to be used by humans into commercial service without human-interface studies, systemic and field-use-sensitive risk assessment, and possible design revisions.
6. Oppose corporate marketing rhetoric that substantially inflates product capability.
7. Do due diligence to avoid accepting offers of employment from technical startups with cultures of excessive secrecy, threats, frequent firings, surveillance, and unduly siloed information.

## Conclusion

Securing sufficient startup and operating capital, recruiting and retaining qualified technical personnel, meeting product-development milestones and deadlines, fulfilling government regulatory requirements, and stoking product expectations and demand, are among the critical challenges faced by technical startups. As exemplified in the Theranos case, such challenges are apt to arise in the fund-raising, teambuilding, product-development, regulatory and marketing phases of the entrepreneurial stage of the overarching R&I process.

The urgent need to meet such challenges and the major financial stakes often involved suggest that scientists and engineers in technical startups should be prepared to encounter situations that press them to engage or acquiesce in ethically irresponsible conduct. It would be beneficial for potential future startup scientists and engineers to explore the Theranos case during their studies. Doing so would convey the cautionary message that, under managerial pressure, technical professionals in startups sometimes resort to ethically irresponsible conduct.

However, more in line with this paper’s focus, such study would also impart an important constructive message: notwithstanding strong workplace pressures, startup scientists and engineers sometimes show remarkable determination, courage, and resourcefulness in sustaining ethically responsible work practice. It is in society’s best interest for scientists and engineers who work in technical startups and who may be involved in meeting their characteristic challenges to be equipped to act in ethically responsible ways. Familiarity with the FERSEs and knowledge of the kinds of ethical challenges, ethically responsible actions, and harm-prevention strategies exhibited in the Theranos case can help bring that goal closer to realization.

## Footnotes

1 See, for example, Resnik (1998), Committee on Science, Engineering, and Public Policy (2009), Shamoo and Resnik (2015), and Iphofen (2020).

2 See, for example, Harris, Sapienza, and Bowie (2009).

3 Carreyrou (2018a).

4 Alex Gibney’s documentary film, *The Inventor: Out for Blood in Silicon Valley* (TI), is also a useful resource. It contains interviews with three Theranos scientists and engineers not discussed in BB.

5 Other Theranos scientists and engineers engaged or acquiesced in conduct that is arguably ethically irresponsible.  

6 The two published sources on which this paper relies most are Carreyrou’s *Bad Blood* and the author’s *The Ethical Engineer*. The overview of the Theranos episode in Section II and the mini cases in Section IV incorporate many factual claims drawn from *Bad Blood*. The framework of fundamental ethical responsibilities of scientists and engineers sketched in Section III and used in Section IV is discussed in detail in Chapter 3 of *The Ethical Engineer.*

7 TI, 07:20.

8 Auletta (2014).

9 BB, p. 14.

10 *Ibid*., p. 218.

11 *Ibid*., p. 14.

12 Auletta (2014).

13 “Holmes, Balwani, and Theranos raised more than $700 million from late 2013 to 2015…” U.S. Securities and Exchange Commission (2018), p. 2.

14 Robertson became an advisor to Theranos and a member of its Board of Directors in 2004. (Auletta, 2014). However, when a newspaper article critical of the firm was published in October 2015, its Board had slim technical expertise relevant to blood testing science and technology. The Board members at that point were George Shultz, Gary Roughead, William Perry, Sam Nunn, James Mattis, Richard Kovacevich, Henry Kissinger, Riley Bechtel, William Frist, William Foege, Elizabeth Holmes, and Ramesh Balwani. Four of them had some sort of technical background: Perry (PhD, mathematics), Kovacevich (MS, industrial engineering), Frist (MD and heart and lung surgeon), and Foege (MD). See Pflanzer (2015).

15 BB, p. 226.

16 *Ibid*., p. 166. In January 2010, Theranos emailed Walgreens “stating that it had developed small devices capable of running any blood test from a few drops pricked from a finger in real time and for less than half the cost of traditional laboratories.” (*Ibid*., p. 83). However, in 2015, Theranos’s clinical lab director explained to Carreyrou that “of the more than 240 tests Theranos offered in its menu, only about 80 were performed on small finger-stick samples (a dozen on the Edison and another 60 or 70 on the hacked Siemens machines). The rest, he’d said, required…the dreaded hypodermic needle.” (*Ibid*., p. 234).

17 Centers for Medicare & Medicaid Services (2021).

18 BB, p. 188.

19 *Ibid*., p. 186.

20 MLO Staff (2013).

21 BB, p. 185.

22 *Ibid*., p. 190.

23 Mole (2016).

24 BB, p. 168. Nevertheless, some Walgreens patient samples were obtained via venipuncture and normal syringes. They were processed in a Theranos testing lab in Arizona equipped with commercial analyzers. (*Ibid*., p. 293).

25 *Ibid.*, p. 281.

26 *Ibid*., pp. 281–283.

27 *Ibid*., p. 286.

28 Carreyrou (2015).

29 Carreyrou (2018a), p. 287.

30 *Ibid*., p. 289.

31 *Ibid*., pp. 292–293.

32 Alltucker (2016a).

33 Alltucker (2016b).

34 Carreyrou (2018b).

35 U.S. Securities and Exchange Commission (2018).

36 U.S. Attorney’s Office, Northern District of California (2021).

37 Griffith (2021).

38 U.S. Attorney’s Office, Northern District of California (2022a).

39 U.S. Attorney’s Office, Northern District of California (2022b).

40 Ibid.

41 The reason an ethical framework for engineers (and scientists) is important is that without one, ethical judgments about professional conduct often amount to rhetorically embellished expressions of personal feeling or subjective intuition. Without such a framework, the resolution of disagreements over the ethical acceptability of scientists’ and engineers’ conduct may hinge on whether disputants’ personal feelings or subjective intuitions about that conduct agree or disagree. This would preclude rational, evidence-based discussion and could tempt groups whose members share the same feeling or intuition about the ethical acceptability of practitioner conduct to impose their judgments on those who don’t. General, empirical evidence-based ethical frameworks facilitate anchoring judgments about the acceptability of practitioner conduct on factors other than personal feeling or subjective intuition, such as projected harms, benefits, and risks for all affected parties. Disagreements over ethical judgments about the ethical acceptability of practitioner conduct that stem from differences over factual matters can be discussed, debated, and possibly resolved in a way that disagreements over such judgments that stem from divergent personal feelings and subjective intuitions cannot.

42 The Fundamental Ethical Responsibilities of Engineers (FEREs) are discussed in detail in Chapter 3 of the author's  *The Ethical Engineer*. Since scientists and engineers have the same *fundamental* ethical responsibilities, the detailed account of the FEREs presented in Chapter 3 of *The Ethical Engineer* also applies to the FERSEs listed in this section.

43 An anonymous reviewer of an earlier version of this paper commented that “in some ways the value of the framework is being tested alongside the qualities (principles/values) of the [mini] ‘cases’.” There is a sense in which the mini cases 'test' the value of the FERSE framework. Indeed, if there was a clear example in one of the mini cases of conduct by a scientist or an engineer that was consensually deemed ethically irresponsible but that was in accord with all the FERSEs, or that was consensually deemed ethically responsible but that violated one or more of the FERSEs, that would raise doubts about whether the FERSE framework is a reliable resource for assessing whether conduct by practicing scientists and engineers is ethically responsible.

44 For the purposes of this paper, FERSE2 is arguably the most important FERSE.

45 FERSE4 replaces an idea long found in many professional engineering society codes of ethics: that engineers have a primary ethical responsibility to be loyal to their employers and clients. Indeed, the National Society of Professional Engineers’ current Code of Ethics contains much the same idea. Its Fundamental Canon I.4 reads, “Engineers in the fulfillment of their professional duties, shall act for each employer or client as faithful agents or trustees.” (National Society of Professional Engineers, 2019

46 Some ethics scholars’ notions of “others” also include “wildlife” and “ecosystems” on which the survival of human and non-human lives depends.

47 Illegitimate interests that some employers have promoted on occasion include stealing a competitor’s intellectual property, bringing a risky product to market without adequate safety testing, and cheating in satisfying applicable government regulations that affect when a new company product can be released to market.

48 The bulk of the details included in this section’s mini cases were drawn from *Bad Blood*.

49 BB, pp. 30–31.

50 *Ibid*., p. 34.

51 *Ibid.*, p. 37.

52 *Ibid*.

53 Ibid., p. 33.

54 Ibid., p. 34.

55 Together with Theranos engineer Mike Bauerly. See *ibid.,* p. 45.

56 *Ibid*.

57 *Ibid*., p. 46.

58 *Ibid*., p. 53.

59 *Ibid*.

60 *Ibid*., p. 32.

61 Ibid., p. 52.

62 *Ibid*., p. 52.

63 Interestingly, Maxwell’s resignation email included a statement that evokes FERSE4: “I feel like I owe you this bad attempt at an exit interview…” See *ibid*., p. 53.

64 *Ibid*., p. 174.

65 *Ibid*., pp. 186–187.

66 TI, 1:19:14–34.

67 *Ibid*., p. 187.

68 *Ibid.*

69 *Ibid*.

70 *Ibid*., p. 193.

71 *Ibid*., p. 194.

72 *Ibid.*

73 *Ibid*., p. 195.

74 *Ibid*., p. 196.

75 *Ibid*., p. 197.

76 See McGinn (2018), pp. 157–159, for discussion of the conditions under which a scientist’s or engineer’s fundamental ethical responsibility to try to prevent harm or unreasonable risks of harm implies the more specific ethical responsibility to engage in public whistleblowing in trying to do so.

77 BB, p. 185.

78 *Ibid*., p. 188.

79 *Ibid*.

80 *Ibid*., pp. 188–189.

81 Randazzo (2021a, 2021b).

82 Dorothy Atkins (2021).

83 BB, p. 198.

84 *Ibid*.

85 *Ibid*.

86 *Ibid*., pp. 199–200, and Randazzo (2021a, 2021b).

87 *Ibid*., p. 281.

88 Randazzo et al., (2021a, 2021b).

89 In 2019, Erika Cheung co-founded a non-profit organization devoted to “providing investors, entrepreneurs and workers with resources to better recognize and manage ethical issues in emerging companies.” See Ethics in Entrepreneurship (2021).

90 BB, p. 114.

91 *Ibid*., pp. 115–116.

92 *Ibid*., p. 142.

93 *Ibid*.

94 In an email to the author on 3 November 2021, Gangakhedkar confirmed that she was the individual referred to in BB as “Anjali Laghari.”.

95 BB, p. 171.

96 *Ibid*., p. 172.

97 Rosenblatt (2021a).

98 Renda (2021).

99 *Ibid*..

100 Gangakhedkar email to the author on 10 November 2021.

101 Carreyrou confirmed that Rosendorff was referred to in BB as “Alan Beam.” See https://twitter.com/johncarreyrou/status/1442983415196815361. Accessed November 1, 2021.

102 BB, p. 214.

103 *Ibid*., p. 215.

104 *Ibid*., p. 218.

105 Baron (2021b).

106 Somerville (2021c).

107 *Ibid*.

108 BB, p. 216.

109 *Ibid*., p. 221.

110 *Ibid*., p. 230.

111 Randazzo (2021b).

112 BB, p. 214.

113 The two ethically responsible technical practitioners highlighted in the mini cases who did not resign were fired (MC6, MC7).
